# HA PEGylated Filler in Association with an Infrared Energy Device for the Treatment of Facial Skin Aging: 150 Day Follow-Up Data Report

**DOI:** 10.3390/ph15111355

**Published:** 2022-11-02

**Authors:** Paweł Kubik, Jerzy Jankau, Raffaele Rauso, Hassan Galadari, Marina Protasoni, Wojciech Gruszczyński, Dariusz Grzanka, Marta Smolińska, Paulina Antosik, Maria-Luiza Piesiaków, Lidia Kodłubańska, Anna Zagajewska, Bartłomiej Łukasik, Giorgio Stabile, Nicola Zerbinati

**Affiliations:** 1 Centrum Medyczne dr Kubik, Skwer Kościuszki 15/17, 81-370 Gdynia, Poland; 2 Department of Plastic Surgery, Medical University of Gdansk, 80-210 Gdańsk, Poland; 3 Maxillofacial Surgery Unit, University of Campania “Luigi Vanvitelli”, 80138 Naples, Italy; 4 Department of Medicine, College of Medicine and Health Sciences UAE, University Al Ain, Al-Ain 15551, United Arab Emirates; 5 Department of Medicine and Surgery, University of Insubria, 21100 Varese, Italy; 6 Department of Clinical Patomorphology, Nicolaus Copericus University, 85-094 Bydgoszcz, Poland; 7 Centro Medico Polispecialistico, 27100 Pavia, Italy; 8 Department of Clinical Dermartology, Università Vita-Salute San Raffaele, 20132 Milan, Italy

**Keywords:** HA PEGylated filler, PEG-cross-linked hyaluronic acid, infrared energy device, facial skin aging

## Abstract

Background: The face is the area most exposed to the normal course of skin aging, both intrinsically and extrinsically. The aim of the study was to evaluate the cellular and clinical response of a therapeutic protocol aimed at countering facial skin aging. Materials and Methods: Twenty female patients with facial skin laxity and photodamage underwent combined therapy including mesotherapy using non-cross-linked hyaluronic acid with calcium hydroxyapatite and an infrared energy-based device treatment with subsequent implementation of PEG-cross-linked hyaluronic acid soft tissue fillers. To evaluate the benefits, patients underwent histological, immunological, and biomechanical evaluations before the treatment and at 21 and 150 days after the treatment. Results: The histological results at 21 days and 150 days after the procedure showed an increase in the number of fibroblasts and angiogenesis. As for the immunological aspect, it was shown that the treatment has an immunomodulating action, avoiding the activation of CD4 and CD8 cells. Biomechanical data showed that, at 150 days after treatment, the average changes in skin elasticity increased by 72% and the skin hydration increased by 49%. Conclusions: A combination of an infrared energy-based device treatment with both non-cross-linked hyaluronic acid and novel PEG-cross-linked hyaluronic acid leads to numerous positive cutaneous changes after histological, immunological, and biomechanical evaluations.

## 1. Introduction

Intrinsic skin aging represents the normal course of aging for facial tissues [1], whereas extrinsic aging is mainly caused by some factors [2], such as exposure to UV radiation, pollution, and cigarette smoking. The face area is more exposed than other areas and is therefore subjected to both types of skin aging. Skin glow and skin texture have a significant impact on attractiveness. For the prevention or early treatment of superficial skin laxity and/or fine wrinkles, options include topical skincare (e.g., UV protection, antioxidants, retinoids, moisturizers, and DNA repair enzymes), smoking abstinence or cessation, and some device-based rejuvenating procedures [3,4,5,6].

### 1.1. Hyaluronic-Acid (HA)-Based Fillers

Nowadays, evidence shows that hyaluronic-acid (HA)-based fillers are important, safe, and effective tools that can correct the skin aging process of patients’ faces [7,8]. HA is natively present in the connective tissues of the skin, and its amount decreases with age [9]. HA fillers, which are increasingly used in nonsurgical facial rejuvenation treatments, are glycosaminoglycan complex sugars, comprising alternating D-glucuronic acid and N-acetyl-D-glucosamine units [10,11]. Several HA manufacturers have used proprietary crosslinking technologies to develop portfolios of HA fillers suitable for a variety of indications and injection depths. Additionally, energy-based devices are important tools used daily in aesthetic medicine to correct the skin aging process of patients’ faces [12] but aesthetic physicians have increasingly shown the effectiveness of HA fillers combined with energy-based devices [13,14].

### 1.2. Infrared Radiation (IR) Device Used in the Study

In the case of skin that has manifested a loss of firmness as a result of the aging process, the technology of thermolifting used in combination with infrared radiation (IR) involves hormonal changes and weight change as one of the non-invasive lifting methods. The Zaffiro Z200NG device has a special handpiece finished with sapphire glass, which enhances the emission of infrared light, leading to a thermal effect on the skin. The device emits radiation in the wavelength range of 750–1800 nm to obtain energy density in the range of 5–75 J/cm^2^. During the treatment, we obtain the heating of deep layers of the skin to the temperature of 65 °C. Zaffiro Z200NG is also equipped with water peeling technology, a two-phase stream of air and water, which thoroughly cleanses the skin, enabling the delivery of active ingredients to the skin as well as a better conduction of temperature. Zaffiro Z200NG is a CE-certified device, as a class IIa, rule 9 medical device.

This study’s purpose was to evaluate the cellular and clinical response of a therapeutic protocol providing a synergic administration of unique polyethylene glycol (PEG) cross-linked HA fillers and near-infrared energy-based devices after a 150 day follow-up. This work shows long-lasting results and the influence of individual elements of therapy.

## 2. Results

Twenty female patients with facial skin laxity and photodamage underwent combined therapy with cutometric measurements following the therapy, as the authors’ main issue was to evaluate changes in skin elasticity and hydration, which was determined by problem selection (skin laxity).

Additionally, three of those twenty patients were selected for detailed histological examinations to determine the mechanisms behind the changes developing after the treatment. Histopathological samples taken from the treatment areas were used to evaluate changes in inflammatory infiltration (generally using H&E staining (presence of mononuclear cells) detailed by immunohistochemistry: CD68, CD20, CD8) and tissue rejuvenation detailed by neoangiogenesis (CD31), Vimentin presence, and collagen Masson’s trichrome staining. Histopathological tests support the cutometric tests.

### 2.1. Hematoxylin and Eosin Staining

#### 2.1.1. Mononuclear Cells

**Figure 1:**
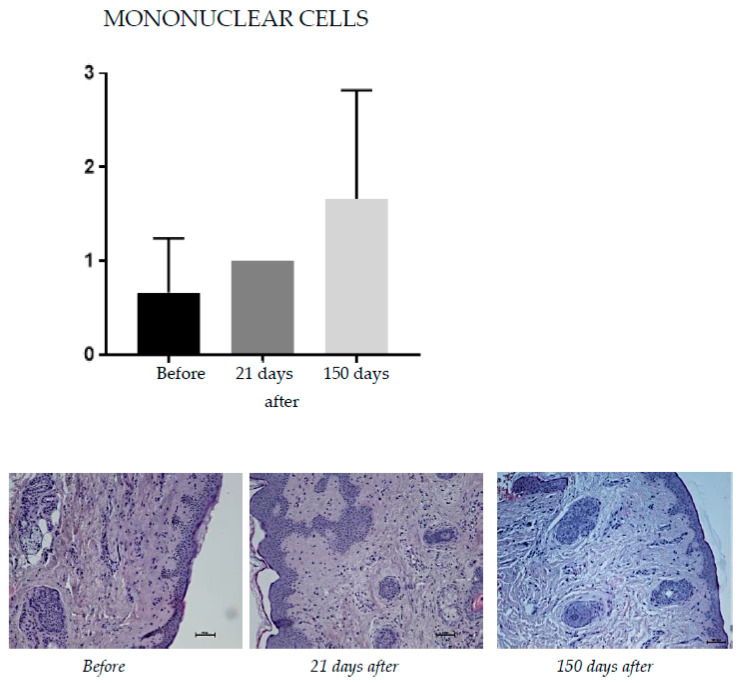
Patient, 61 years old. Hematoxylin and eosin staining, from **left** to **right**: before treatment; 21 days after treatment; 150 days after treatment.

Hematoxylin and eosin staining was used to assess the tissue’s overall structure by contrast staining of the cytoplasm and cell nuclei. Inflammatory infiltration was assessed on a scale of 0–4 to evaluate mononuclear cell infiltration (0—not found; 1—constitute up to 25% of inflammatory infiltrate; 2—constitute from 26–49% of inflammatory infiltrate; 3—constitute from 50–75% inflammatory infiltrate; 4—from 76–100% of inflammatory infiltrate) (Figure 1).

#### 2.1.2. CD68 (Monocytes and Macrophages)

**Figure 2:**
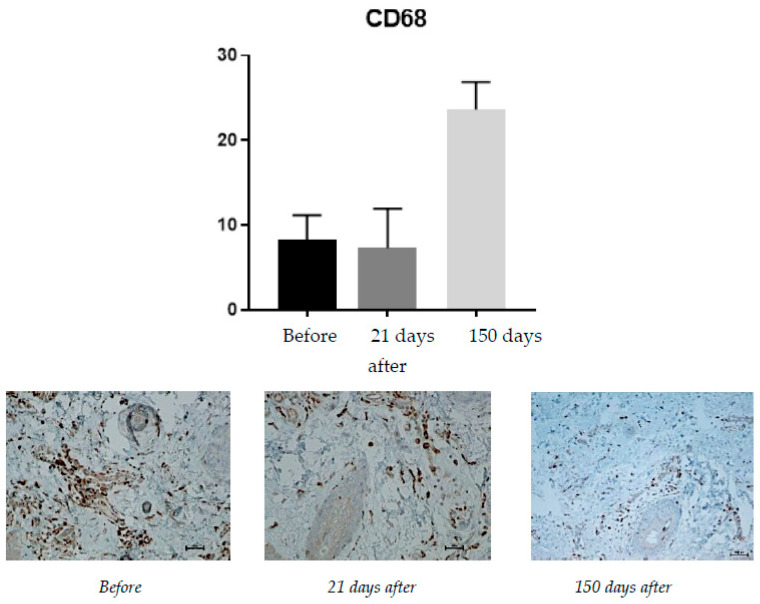
Patient, 61 years old. Comparison of CD68 expression, from **left** to **right**: immediately before the procedure; 21 days after the procedure; 150 days after the procedure.

Protein expression was rated on a scale of 0–100% of inflammatory infiltrate cells (Figure 2).

#### 2.1.3. CD20 (B Lymphocytes)

**Figure 3:**
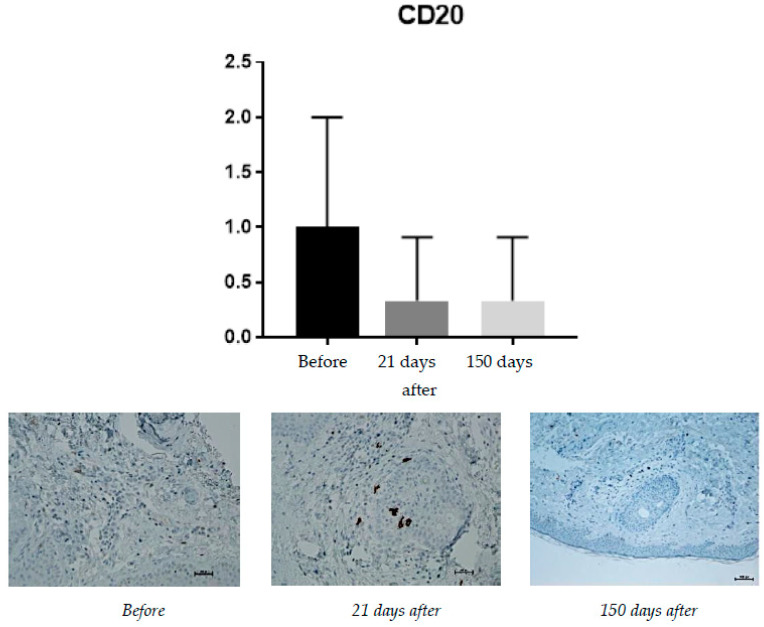
Patient, 60 years old. From **left** to **right**: before treatment; 21 days after treatment; 150 days after treatment.

Expression of CD20 is associated with the presence of B lymphocytes, responsible for recognizing antigens without the involvement of the major histocompatibility system and for the production of antibodies and cytokines. Hence, the presence of B lymphocytes may have a significant impact on the occurrence of inflammatory reactions after procedures, including the administration of fillers based on hyaluronic acid. The increase in their percentage should be negatively considered in the context of the safety and durability of the procedures (Figure 3).

#### 2.1.4. CD8 (T Lymphocytes)

**Figure 4:**
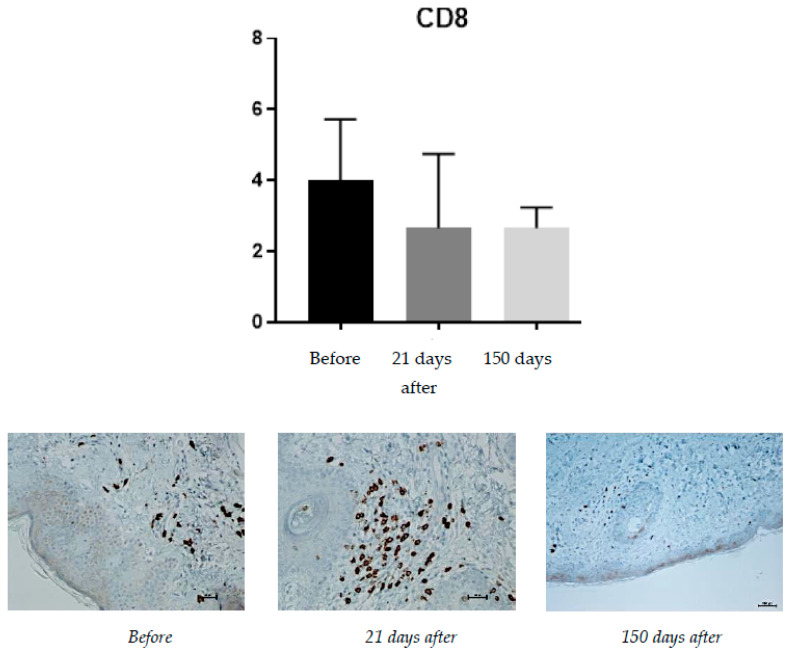
CD8 expression, 61-year-old patient. From **left** to **right**: condition before treatment; condition 21 days after treatment; condition 150 days after treatment.

Protein expression was rated on a scale from 0–100% of inflammatory infiltrate cells (Figure 4).

#### 2.1.5. CD31 (PECAM-1)

**Figure 5:**
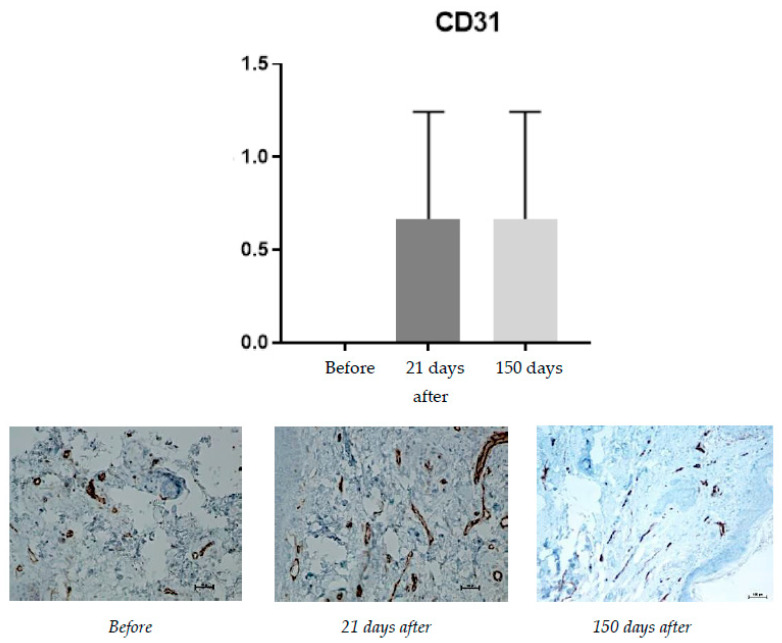
Increase in CD31 expression, from **left** to **right**: before treatment; 21 days after treatment; 150 days after treatment.

CD31 (PECAM-1: Platelet endothelial cell adhesion molecule) is a factor associated with vascular endothelium and its occurrence is associated with angiogenesis. Angiogenesis, along with neocollagenesis, is one of the expected effects of tissue revitalizing procedures. The number of vessels (CD31) was evaluated using a scale of 0–2 (0—few, thin-walled; 1—increased number of vessels with slight wall thickening; 2—increased number of vessels with pronounced wall thickening) (Figure 5).

#### 2.1.6. Vimentin (Presence of Fibroblasts)

**Figure 6:**
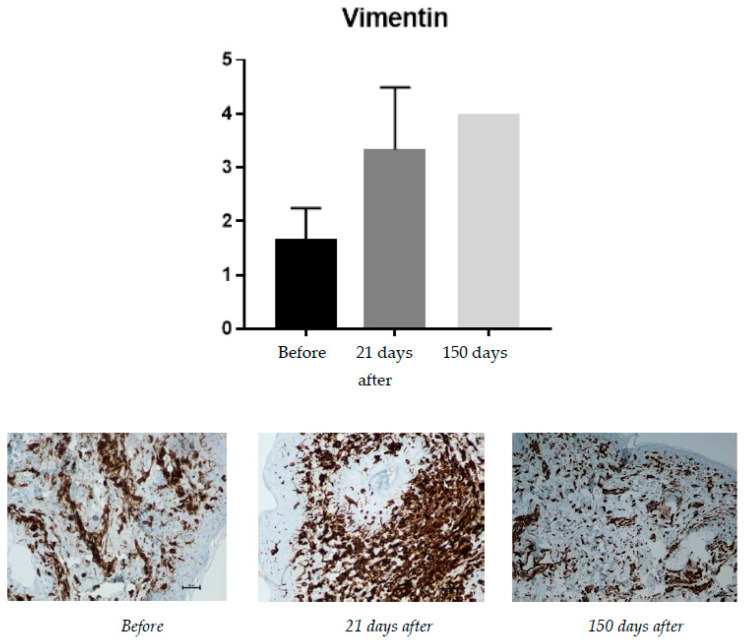
Increase in fibroblasts, from **left** to **right**: before treatment; 21 days after treatment; 150 days after treatment.

The presence of fibroblasts is the strongest evidence of ongoing tissue regeneration. Fibroblasts actively produce collagen, intercellular matrix fibers, and proteoglycans. The presence of fibroblasts in the tissue is evidence of the ongoing process of producing new collagen fibers (Figure 6). To assess fibroblasts presence (Vimentin), a semiquantitative scale (0–4) was used.

#### 2.1.7. Masson’s Trichrome Staining

**Figure 7:**
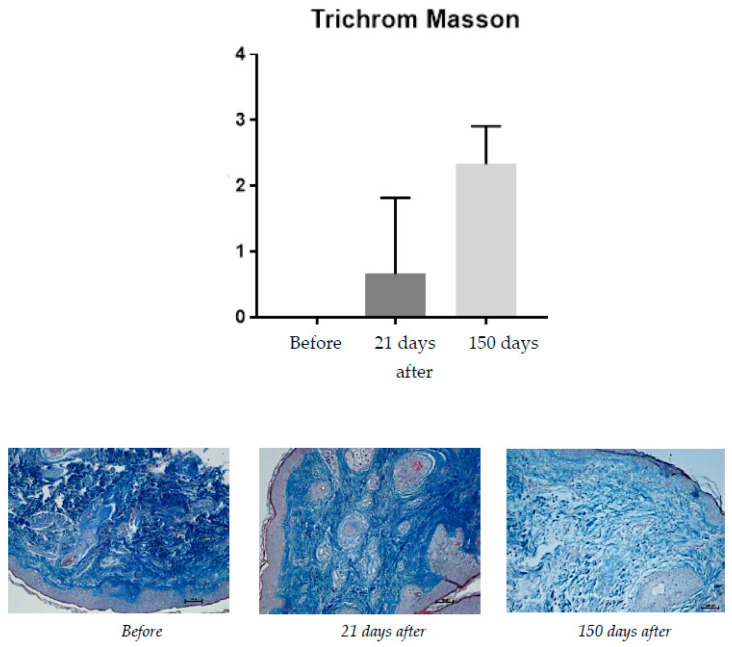
Patient, 61 years old. Masson’s trichrome staining, from **left** to **right**: before treatment; 21 days after treatment; 150 days after treatment.

This staining was used to assess collagen on a scale of 0–3 (0—loose, regular; 1—loose, irregular; 2—concentrated; 3—compact, thick fibers) (Figure 7).

### 2.2. Cutometric Results

Cutometric measurements of skin elasticity and hydration were performed before treatment and at 5 months and 21 days after the treatment. For both elasticity and hydration, a scale of 0–100 (0—worst, 100—best) was used.

**Figure 8:**
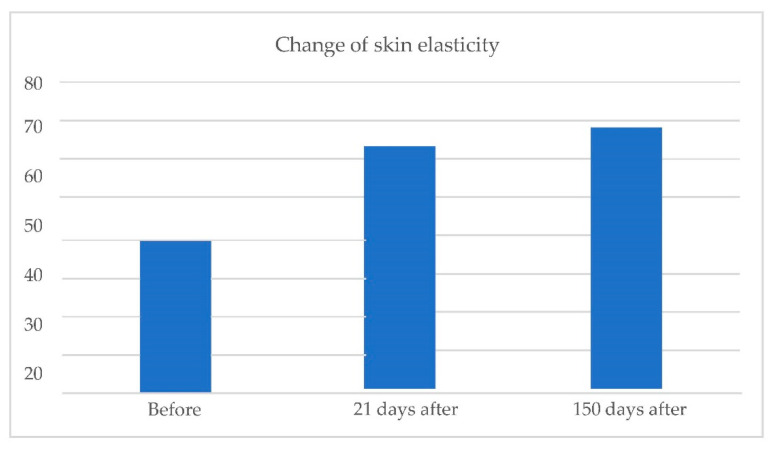
From **left** to **right** change in skin elasticity before treatment; 21 days after treatment; 150 days after treatment.

- Skin elasticity:

Change in skin elasticity (Figure 8).

**Figure 9:**
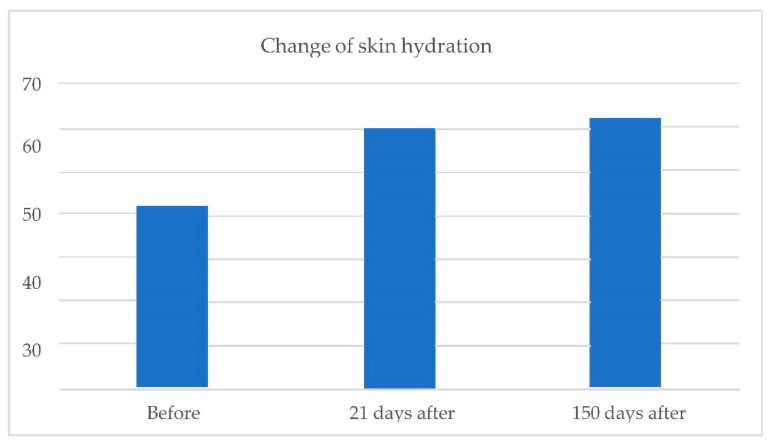
From **left** to **right** change in skin hydration before treatment; 21 days after treatment; 150 days after treatment.

- Skin hydration:

Change in skin hydration (Figure 9).

## 3. Discussion

Twenty female patients underwent combined therapy, including mesotherapy using non-cross-linked hyaluronic acid with calcium hydroxyapatite and an infrared energy-based device treatment with subsequent implementation of PEG-cross-linked hyaluronic acid soft tissue fillers.

### 3.1. Immunohistochemical Staining Results

Hematoxylin and eosin staining was used to assess the tissue’s overall structure, by contrast, staining of the cytoplasm, and cell nuclei. At 150 days after the procedure, an increase in inflammatory infiltration was noted (from 0.66 before treatment to 1.00 at day 21 to 1.68 at 150 days after the procedure).

The expression of CD8 proteins is associated with the presence of cytotoxic T lymphocytes. The CD8 protein is directly associated with the MHC class I recognition system. The increased presence of cytotoxic T lymphocytes (expressed by increased expression of CD8 proteins) is an undesirable effect of the presence of implants, such as hyaluronic-acid-containing fillers. Before the treatment, the average level of CD8 expression was 4% of the inflammatory infiltrate. At 21 days after the procedure, the level of CD8 positive cells decreased to an average of 2.66% and no change was found on the 150th day after the procedure.

CD68 expression is correlated with the number of monocytes and macrophages present, i.e., cells that absorb foreign substances. An increase from 7.33% on day 21 after the procedure to 24.6% at 150 days after the treatment was observed.

Expression of CD20 is associated with the presence of B lymphocytes, responsible for recognizing antigens without the involvement of the major histocompatibility system, and the production of antibodies and cytokines. Before the treatment, the average level of CD20 positive cells was found to be 1% of inflammatory cells. It was expected to reach 0.33% on the 21st day and did not change till day 150.

CD31 (PECAM-1: Platelet endothelial cell adhesion molecule) is a factor associated with vascular endothelium, and its occurrence is connected with angiogenesis. Angiogenesis, along with neocollagenesis, is one of the expected effects of tissue revitalizing procedures. Before the procedure, the level of CD31 was found to be 0 (few, thin-walled vessels); it increased, reaching an average of 0.66 on day 21 and stayed at the same level at day 150.

Vimentin is correlated with the presence of fibroblasts. Before treatment, the average presence of fibroblasts was at 1.66, and it successively grew, reaching 3.33 at day 21 and 4.00 at 150 days after the procedure.

For Masson’s trichrome staining, a change from 0 before the procedure to 0.66 at 21 days and 2.33 after 150 days was observed.

The histological results after 21 days and 150 days after the procedure showed an interesting cause and effect relationship (an increase in the number of fibroblasts, angiogenesis [12,14], and the inflammation in the tissue). This response is expected, both due to the tissue’s traumatization associated with the procedure (a combination of skin damage and high temperature), as well as the introduction of a foreign body (hyaluronic-acid-based fillers).

While the inflammatory process was shown to slightly increase after the procedure, the expression levels of CD8 (T cells) and CD20 (B cells) reduced and did not change at day 150.

This phenomenon must be induced by the hyaluronic-acid-based fillers introduced into the tissue. The compositions of the fillers used here include water, hyaluronic acid, polyethylene glycol, and an admixture of glycine and L-proline [15,16,17]. Based on our observations and other studies, we can conclude that the PEG used in these products (used as a cross-linking agent) has immunomodulatory activity, limiting the inflammatory response at the application site [18].

This observation after 150 days is in line with the discussion we made having the preliminary results after 21 days after the procedure, which is of note from the perspective of the risks associated with the long-term residence of a foreign body, such as a hyaluronic-acid-based filler implant (granulomas and other immune reactions).

The expression of CD68 at 150 days after the procedure was increased vs. day 21; of interest is the observation that, in vivo, hyaluronic acid is depolymerized into lower molecular weight fragments by enzymatic digestion and recent findings support the thesis that hyaluronic acid and its degradation products are important regulators of dendritic cells and macrophages [19], which is in line with our observations.

### 3.2. Cutometric Results

Before the treatment, the average level of skin elasticity (on a scale of 0–100) was 39.36. It increased to 63.27 on day 21 and 68.20 at 150 days after the procedure.

The average level of skin hydration (on a scale of 0–100) before the treatment was 41.73. After treatment, levels increased, achieving a level of 60.50 on day 21 and 62.00 at 150 days after the procedure.

At 150 days after treatment, average changes in skin elasticity increased by 72%, and the skin hydration increased by 49%. The result of the skin hydration increase was probably caused by both mesotherapy and soft tissue filler implementation. We could not find any mechanism that could explain the increase in the amount of skin hydration caused by the infrared energy-based device treatment, either short term or long term. The improvement in skin elasticity can be the result of both infrared treatment and the skin stimulation associated with the implementation of stabilized hyaluronic acid, as well as the interaction of both of these factors.

The cutometric results show a significant effect of the combination therapy on the skin’s elastic properties, combined with a permanent increase in skin hydration, visible even at 150 days after the treatment. The hypothesis we put forward by presenting the data 21 days after the procedure (that PEGylated HA injections in combination with the IR treatment induced the synthesis of dermal components, such as elastin and collagen, which may be responsible for the persistence of this effect) is visible for the data obtained after 150 days. The activation of the collagen synthesis and elastin fibers and their potential renewal may also explain the influence of HA on the skin’s elastic parameters. The newly formed fibers may be more susceptible and their greater number and entanglement may strengthen the collagen and elastin network embedded in the proteoglycan-glycosaminoglycan gel, forming the dermis [20].

Based on the preliminary data obtained after 21 days—the synergistic effect of combination therapy—we also showed that the stimulating effect in terms of skin hydration and elasticity is confirmed by the data 150 days after the treatment.

Cutometric measurements performed in the combination therapy group were significantly higher compared to filler monotherapy and IR monotherapy:

Filler monotherapy:

- Before the treatment, the average level of skin elasticity (on a scale of 0–100) was 57.70. It increased to 66.90 on day 21 and 59.030 at 150 days after the procedure.
- The average level of skin hydration (on a scale of 0–100) before the treatment was 56.10. After treatment, levels increased, achieving a level of 65.80 on day 21 and 67.07 at 150 days after the procedure.

IR monotherapy:

- Before the treatment, the average level of skin elasticity (on a scale of 0–100) was 55.93. It increased to 69.83 on day 21 and 72.90 at 150 days after the procedure.
- An average level of skin hydration (on a scale of 0–100) before the treatment was 54.53. After treatment, levels increased, achieving a level of 56.83 on day 21 and 57.83 at 150 days after the procedure.

We have not found a publication in the literature to which we could refer to the obtained results. Comparison with the existing data on HA mesotherapy [20] proves that the effect of hyaluronic acid (non-cross-linked) is the only, though inadequate, point of reference. The effect of HA mesotherapy on skin elasticity was maintained 3 months after the end of the mesotherapy session, with a decrease in parameters after 3 months, while the control did not significantly affect the measured parameters.

Moreover, compared to the control, the HA effect was more lasting. The HA effect’s remanence was also significant for the entanglement of collagen fibers, with a 10.5% increase in the equivalent strain parameter at 3M compared to D0 (*p* = 0.002), while the control effect was no longer significant. However, the difference in the equivalent strain parameter between HA and control treatments was not significant. The temporal effect of HA control and treatment on dissipation was not significant [20].

In this study, HA was complexed with PEG through a process of cross-linkage of the two polymers (PEGylation), forming a 3D molecular scaffold for a better integration with the connective tissue components, a long-lasting filling effect, and a better resistance of the skin to thermal and mechanical stress. On the basis of our observations, the filler used in this study is safe; it does not show signs of stimulation of the immune system and/or any other adverse reactions. The advantageous properties of PEG, such as its hydrophilicity, non-toxicity and non-immunogenicity, are transferred to the combined filler used here through PEGylation, acting with a supplementary protective action towards the risk of immunological adverse reactions. The injected filler appears harmoniously integrated with the structures inside the connective tissue, as collagen fibers, blood and lymphatic vessels, glands, and nerves.

Due to PEGylation, the filler used here presented excellent rheological properties, such as cohesivity, viscoelasticity, and plasticity, with an optimized adaptation to the receiving anatomic area and, at the same time, it maintained the desired shape for a long-lasting esthetical correction.

## 4. Materials and Methods

Twenty female patients aged 33 to 62 (average 46), with skin phototype I-IV and facial skin laxity and photodamage, were entered into the study. Exclusion criteria included [21]: taking oral and/or local retinoids up to six months prior to the study, excessive tanning, active skin and connective tissue diseases characterized by photosensitivity (e.g., systemic lupus, collagenopathy, or skin porphyria), active herpes simplex infection, taking drugs and/or photoreactive cosmetics (including tetracycline antibiotics and immunosuppression, i.e., cortisone and its derivatives or anticoagulants, i.e., Dipyridamole and coumarin derivatives, cosmetics containing thyme extract, or herbs containing St. John’s wort) for up to six month prior to the study, diseases with immunodeficiency for reasons of caution (depending on the decision of the qualifying doctors), unregulated diabetes, state after other cosmetics and aesthetic procedures within the area undergoing surgery (depending on the particular procedure performed the decision each time was made by the qualifying doctor), acquired vitiligo or melanin production disorder (i.e., hypermelanosis), tattoos in the area undergoing treatment, or taking anti-inflammatory drugs. Each patient signed an informed consent document.

From these twenty patients that underwent the combined therapy for skin laxity, successfully confirmed by cutometric measurements of skin elasticity, three patients were randomly selected for detailed histopathological tests. Base measurement method was cutometry and histopathological tests were supportive in terms of determining detailed tissue reactions that stand behind the results.

Patients underwent the following protocol:

Day 0: Injection of 2.5 cc of mesotherapy quality HA (Hydro Deluxe, Neauvia non-cross-linked hyaluronic acid (18 mg/mL)) with 0.01% calcium hydroxyapatite, glycine, and l-proline.

Day 7: Treatment with a combined water peeling infrared device (Zaffiro, Neauvia; wavelength range 750–1800 nm; fluence of 35–45 J/cm^2^) to the entire face. Therapy was then followed (directly after IR procedure) by treatment with the following facial PEGylated HA filler:

Neauvia Stimulate PEG-cross-linked hyaluronic acid (26 mg/mL) with 1% calcium hydroxyapatite and glycine and I-proline addition; malar area—dermal.

Polyethylene glycol polymer (PEG), used as a cross-linking agent in the hyaluronic-acid-based fillers tested in this study, creates the so-called PEGylation [22,23] effect, which seemed to offer considerable advantages in terms of the safety and performance of the HA gel.

Both PEG and HA are polymers, and their cross-linkage allows the creation of matrices with scaffold structure [24,25,26] as a 3D web constituted by interpenetrated knot sand links, thus offering a better integration of the filler into the connective tissue. Furthermore, such a structure would permit the retention and gradual release of molecules that is suitable for skin rejuvenation. HA was complexed with PEG through a process of cross-linkage of the two polymers (PEGylation), forming a 3D molecular scaffold for better integration with the connective tissue components, a long-lasting filling effect, and a better resistance of the skin to thermal and mechanical stress. PEG’s advantageous properties, such as its hydrophilicity, non-toxicity, and non-immunogenicity, are transferred to the combined filler used here through PEGylation, acting with a supplementary protective action towards the risk of adverse immunological reactions [27]. Due to PEGylation, the filler presents excellent rheological properties, such as cohesivity, viscoelasticity, and plasticity, with an optimized adaptation to the receiving anatomic. The filler provides volume and support to the connective tissue as an injected substance but due to the very high content of polar groups and the molecular structure, it also has a positive associated effect linking an extraordinary quantity of water molecules [28]. In this way, high hydration of the extracellular matrix of the connective tissue is increased and maintained, thus enhancing extracellular matrix permeability and the diffusion of nutrients from blood vessels to the whole skin as an organ, epidermis included, in a renewed young homeostatic balance.

Patients had biopsies taken for histological evaluation. The whole procedure was completed in the near-ear area, where the biopsies were taken. The incisions were performed on different sides on different days to avoid inflammatory reactions connected to the healing process after a previous biopsy. The biopsies were conducted by full surgical excision of the skin, at a diameter of about 1 cm in length.

Sections for histological tests were taken before the treatment at 21 and 150 days after the treatment. Skin biopsies were fixed in 10% buffered formalin at pH = 7.2 for further evaluation. The tissue block was cut into thick sections (5 μm) for histological examinations and into sections of 3–4 μm for immunohistochemical examinations. Hematoxylin and eosin (H&E) staining was used to evaluate the cellular content and general histoarchitecture of tissue specimens. Masson’s trichrome stain was used to evaluate the degree of preservation of collagen and elastic fibers in facial skin tissue.

The immunohistochemical staining was performed using mouse monoclonal anti-CD68 (KP-1) antibody, rabbit monoclonal anti-CD8 (SP57) antibody, mouse monoclonal anti-CD20 (L26) antibody, mouse monoclonal anti-CD31 (JC70) antibody, and mouse monoclonal anti-Vimentin (V9) antibody.

Expression analysis:

Two independent pathologists’ evaluations of protein expression were performed at 10× and 20× the original objective magnification using a light microscope ECLIPSE E400 (Nikon Instruments Europe, Amsterdam, Netherlands). Inflammatory infiltration (H&E stain) was assessed on a scale of 0–4 including mononuclear cells: 0—not found; 1—up to 25% of inflammatory infiltrate; 2—from 26–49% of inflammatory infiltrate; 3—from 50–75% of inflammatory infiltrate; 4—from 76–100% of inflammatory infiltrate. Three-color staining, according to Masson (Special Stain Kit Masson’s Trichrome, DiaPath, Martinengo, Italy), was used to assess collagen on a scale (0—loose, regular; 1—loose, irregular; 2—concentrated; 3—compact, thick fibers). Evaluation of the expression of the CD4 [29], CD8 [30], and CD68 [31] proteins was assessed on a scale of 0–100% of inflammatory infiltrate cells and CD20 [32] on a scale of 0–1. However, the number of vessels (CD31) [33] was assessed using a scale of 0–2 (0—few, thin-walled; 1—increased number of vessels with slight wall thickening; 2—increased number of vessels with pronounced wall thickening). A semiquantitative scale (0–4) was used to assess the presence of fibroblasts-Vimentin [34].

Finally, cutometric measurements of skin elasticity and hydration were also performed before the treatment and at 21 and 150 days after the treatment using Courage + Khazaka Multi Skin Test Center MC 1000.

## 5. Conclusions

This study is the first study that combined near-IR energy devices with injectable products and tried to find out detailed mechanisms of skin improvement, especially in the field of histological changes such as neoangiogenesis (CD-31) and collagen structure change (Masson’s trichrome staining).

The combination of an infrared energy-based device treatment with both non-cross-linked hyaluronic acid and novel PEG-cross-linked hyaluronic acid was shown to lead to numerous positive cutaneous changes after histological, immunological, and biomechanical evaluations.

Combined therapy, both in the short-term and long-term approach after 150 days, changes the skin elasticity by +72% and moisturizes the skin by +49% (the effect after 21 days is +60% elasticity and +45% hydration), which is a very desirable effect from the perspective of both the patient and the doctor. The increase in skin elasticity may be the result of both infrared treatment and skin stimulation associated with the introduction of stabilized hyaluronic acid, as well as the interaction of both factors.

The results are interesting from the point of view of the risks of long-term exposure to a foreign body, such as a hyaluronic acid filler implant (granulomas and other immune responses). According to the authors, this promising observation should be extended and compared with other procedures (in particular, isolated infrared therapy and isolated therapy with products containing hyaluronic acid cross-linked with polyethylene glycol) in order to more accurately determine the course of immune reactions in the presence of polyethylene glycol contained in products placed in patients’ tissues.

In the future, more studies will be required to correlate these changes with clinical findings.

## Data Availability

Not applicable.
